# Remote sensing of the impact of flash drought events on terrestrial carbon dynamics over China

**DOI:** 10.1186/s13021-020-00156-1

**Published:** 2020-09-22

**Authors:** Miao Zhang, Xing Yuan, Jason A. Otkin

**Affiliations:** 1 Key Laboratory of Regional Climate-Environment for Temperate East Asia (RCE-TEA), Institute of Atmospheric Physics, Chinese Academy of Sciences, Beijing, 100029 China; 2 School of Hydrology and Water Resources, Nanjing University of Information Science and Technology, Nanjing, 210044 China; 3 College of Earth and Planetary Sciences, University of Chinese Academy of Sciences, Beijing, 100049 China; 4 Cooperative Institute for Meteorological Satellite Studies, Space Science and Engineering Center, University of Wisconsin-Madison, Madison, WI 53706 USA

**Keywords:** Flash drought, MODIS, Soil moisture, GPP, NPP, LAI

## Abstract

**Background:**

Flash drought poses a great threat to terrestrial ecosystems and influences carbon dynamics due to its unusually rapid onset and increasing frequency in a warming climate. Understanding the response of regional terrestrial carbon dynamics to flash drought requires long-term observations of carbon fluxes and soil moisture at a large scale. Here, MODIS satellite observations of ecosystem productivity and ERA5 reanalysis modeling of soil moisture are used to detect the response of ecosystems to flash drought over China.

**Results:**

The results show that GPP, NPP, and LAI respond to 79–86% of the flash drought events over China, with highest and lowest response frequency for NPP and LAI, respectively. The discrepancies in the response of GPP, NPP, and LAI to flash drought result from vegetation physiological and structural changes. The negative anomalies of GPP, NPP, and LAI occur within 19 days after the start of flash drought, with the fastest response occurring over North China, and slower responses in southern and northeastern China. Water use efficiency (WUE) is increased in most regions of China except for western regions during flash drought, illustrating the resilience of ecosystems to rapid changes in soil moisture conditions.

**Conclusions:**

This study shows the rapid response of ecosystems to flash drought based on remote-sensing observations, especially for northern China with semiarid climates. Besides, NPP is more sensitive than GPP and LAI to flash drought under the influence of vegetation respiration and physiological regulations. Although the mean WUE increases during flash drought over most of China, western China shows less resilience to flash drought with little changes in WUE during the recovery stage. This study highlights the impacts of flash drought on ecosystems and the necessity to monitor rapid drought intensification.

## Background

Flash drought is characterized by rapid development from normal to dry conditions at sub-seasonal timescales [1–4]. The rapid onset of drought leaves limited time for preparation and drought mitigation, causing devastating impacts due to insufficient early warning [5, 6]. The increasing frequency of flash drought in a warming climate [4, 7] presents a higher risk on vegetation health, food security, and ecosystem and environmental sustainability [8, 9]. Extreme drought events could dampen carbon uptake and accelerate atmospheric carbon dioxide concentrations [10–12]. The 2013 summer drought in southern China is a once-a-century drought event with the unusual speed of drought development [6, 13], resulted in a net reduction of 101.5 Tg C in carbon sequestration. Increased understanding of how different ecosystems respond to flash drought onset and recovery is needed to predict the future terrestrial carbon sink and atmospheric CO_2_ concentrations, as well as offer guidelines on the implementation of mitigation and adaptation strategies.

Drought can influence the carbon cycle by altering the physiological functioning including photosynthesis and vegetation respiration. In addition to the direct impacts of drought on the terrestrial carbon sink, leaf area index (LAI) as a measure of vegetation structure is also changed under drought, which in turn influences the carbon cycle. Satellite images offer regional insights regarding terrestrial vegetation conditions [14] and can be used to monitor the ecological impacts of drought [10, 15]. High-resolution vegetation property datasets retrieved from the Moderate Resolution Imaging Spectroradiometer (MODIS) sensor are widely used to monitor the dynamics and characteristics of vegetation [16, 17]. These products provide global 8-day gross primary productivity (GPP), net primary productivity (NPP) based on a light use efficiency model [14], and LAI. The vegetation productivity and LAI show different responses to drought from vegetation physiological and structural perspectives, respectively [18, 19]. NPP is controlled by GPP and vegetation autotrophic respiration. Vegetation respiration could be impaired under severe drought, whereas sometimes it can be enhanced, thereby further increasing carbon loss for some cases of drought [20]. Otkin et al. [12] assessed the evolution of vegetation conditions during the 2012 flash drought over the U.S. They found that vegetation response to the drought conditions was significant and preceded changes in the United States Drought Monitor (USDM) drought depiction. Xie et al. [13] and Yuan et al. [6] assessed the enormous carbon loss during the 2013 summer drought over southern China through eddy covariance and satellite observations of vegetation greenness, as well as modeling.

Ecosystem water use efficiency (WUE) is also influenced by drought [21], which is defined as the ratio of carbon assimilation to evapotranspiration (ET). Drought could induce stomatal closure to reduce water loss, thus decreasing carbon uptake. The response of WUE varies among ecosystems [22, 23] and droughts with different duration and severity [24, 25]. Higher WUE during droughts was found in Northeast China and South China comprised of abundant forests, whereas WUE was decreased over northwestern China [23]. However, how the multiple ecosystem metrics (GPP, NPP, LAI, and WUE) respond to flash droughts and how the response time varies over different regions remain unclear.

In this paper, we address the response of vegetation to flash drought over China using multiple vegetation indices. Using soil moisture (SM) from the European Centre for Medium-Range Weather Forecasts Reanalysis 5 (ERA5) [26], flash drought is identified during 2003–2018, and meteorological conditions during the onset and recovery stages of flash drought are also investigated. In addition, we will examine the response of GPP, NPP, LAI, and WUE to flash droughts using MODIS satellite observations.

## Data and methods

### Data

#### Remote observations of carbon fluxes and LAI

The U.S. National Aeronautics and Space Administration (NASA) produces the global products of GPP and NPP. The Terra MODIS sensor provides 8-day GPP and NPP at 500-m horizontal resolution starting in 2000 [14]. The GPP is formulated by photosynthetically active radiation (PAR), fraction of photosynthetically active radiation (fPAR), and the PAR conversion efficiency determined by land cover type, and meteorological conditions [27] in a light use efficiency model. The subsequent estimation of maintenance and growth respiration is subtracted from GPP to obtain NPP. They improve the estimates of ecosystem productivity and have been widely used in many studies [28, 29]. The 8-day LAI product from 2003 to 2018 is derived from MODIS at 500-m resolution (MCD15A3) and is computed using spectral reflectance from MODIS measurements. The GPP, NPP, and LAI from MODIS products are all aggregated to 0.25-degree resolution.

#### Hydrometeorological variables

The observations of precipitation, temperature, and relative humidity are obtained from more than 2400 Chinese Meteorological Administration meteorological stations, which are subsequently interpolated to a 0.25-degree resolution grid through anomaly approach [30]. The meteorological stations are mainly distributed over eastern China, so the gridded data may have larger uncertainty over western China [30]. However, our previous study [4] suggests that flash droughts over western China are much less frequent than those over eastern China, so the influence of observation uncertainty might be limited. The climatology of in situ meteorological observations is firstly interpolated by thin-plate smoothing splines, and the anomalies of the meteorological variables derived from angular distance weighting method is added to the climatology to obtain the gridded meteorological variables. Temperature and relative humidity are used to calculate vapor pressure deficit (VPD) which has a large impact on WUE through influencing stomatal behaviors [31].

Soil moisture is used to identify flash droughts, which is closely correlated with vegetation dynamics [32]. Due to the lack of in situ observations of soil moisture and ET, the soil moisture and ET over China can be estimated through land surface models. Here, the soil moisture and ET databases are derived from ERA5. ERA5 provides 3-hourly soil moisture and ET at 0.25° resolution started in 1979. Here we use the top 1-m soil moisture aggregated from the top 3 soil layers with depths of 0–7, 7–28, 28–100 cm, where we multiply soil moisture in each layer with weights corresponding to their thicknesses. The study period is from 2003 to 2018 based on the overlapping periods of all datasets.

### Methods

#### Identification of flash drought events

The definition of flash drought is based on soil moisture addressing the rapid intensification of drought [2, 4]. Flash drought starts when the 8-day mean soil moisture declines from above the 40th percentile down to the 20th percentile, with a decreasing rate of no less than 5% in percentile per 8-day period, which is the “onset” stage of flash drought. The evolution from 40th to 20th soil moisture percentiles depicts the drought development. The recovery stage of flash droughts starts when the soil moisture percentile starts to either increase or decrease more slowly. Once the soil moisture recovers to above the 20th percentile, the drought ends. The 20th percentile of soil moisture is selected as the drought threshold according to Yuan et al. [4], which can represent different climate conditions because the 20th percentile thresholds in actual soil moisture vary for different climatological distributions. The duration of a flash drought event is no less than 24 days to exclude short dry spells that have little impact on ecosystems. We focus on April-September during which the growth of vegetation over China usually occurs. We also focus on three sub-regions where flash drought occurs frequently and the ecosystem productivities are concentrated, including southern China (21–32 °N, 98–123 °E), northern China (32–42 °N, 98–126 °E) and northeastern China (42–54 °N, 119–135 °E).

#### Response of ecosystem to flash drought

Ecosystem photosynthesis and respiratory processes can be suppressed when vegetation is under water stress [33] and GPP is the total ecosystem carbon uptake representing the dynamics of vegetation photosynthetic functioning. NPP is the discrepancy between GPP and autotrophic respiration integrating the impacts of drought on photosynthesis and respiration [20]. LAI is the green leaf area per unit ground area, which can be used to detect changes of vegetation state (e.g., defoliation). Photosynthetic CO_2_ uptake is influenced by stomatal conductance, Rubisco activity, and LAI [34]. When the photosynthetic process is suppressed during drought, the vegetation structure may remain unchanged [35]. The occurrences of negative anomalies of ecological metrics are considered as the onset of ecological response. Here, the anomalies of 8-day mean GPP, NPP, and LAI are all standardized according to Eq. (1):

1 $$X_{i,j} = \frac{{X_{i,j} - u_{j} }}{{\sigma_{j} }} ,$$

where *X* is the ecological variable, *i* is the year and *j* is the timing in the *ith* year. $u_{j}$ is the mean of *X,* and $\sigma_{j}$ is the standard deviation of *X,* at the *jth* calendar 8-day computed using data from 2003 to 2018. Here, we use the response time index to investigate the relationship between flash drought and ecological impact [36], which is defined as the timing of first occurrence of negative standardized ecological metrics during flash droughts. The response frequency is the ratio of the flash droughts during which there is a decline in ecological indexes to the total number of flash droughts for each grid. A higher response frequency implies that flash droughts pose a higher risk to the ecosystem. WUE is the ratio of GPP to ET quantifying the trade-off between carbon assimilation and water loss through ET. Water and carbon cycles are coupled through stomata, and the vegetation would take adaptation measures to cope with drought such as increasing its WUE [37]. However, WUE is not only sensitive to drought but also to the dynamics of VPD. Zhou et al. [31] proposed underlying water use efficiency (uWUE) as a way to incorporate the effects of VPD on the relationship between photosynthesis and ET. uWUE is formulated as $\frac{{GPP \times \sqrt {VPD} }}{ET}$, which is supposed to better reflect the linear relationship among GPP, ET, and VPD [31]. It is also related to plant physiological regulation through stomata regardless of the effects of VPD. The variations of uWUE are assumed to be more strongly correlated with drought. However, WUE is also dependent on VPD especially at sub-daily and daily time scales. Both the anomalies of WUE and uWUE are standardized according to Eq. (1).

## Results

### Climatological and ecological characteristics over China

**Fig. 1:**
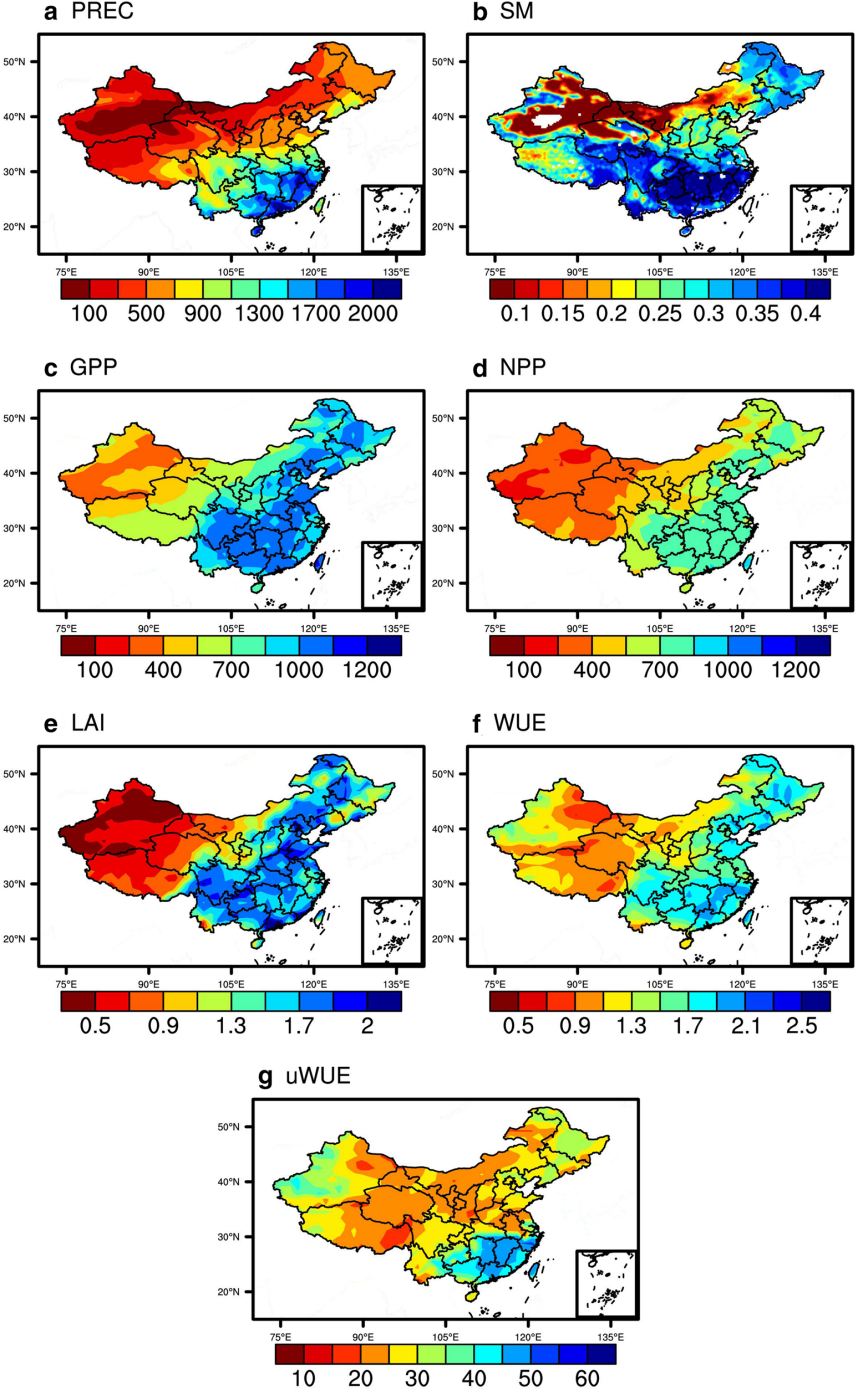
Spatial distribution of annual mean (**a**) precipitation (mm yr^−1^), (**b**) soil moisture (m^3^ m^−3^), (**c**) GPP (g C m^−2^ yr^−1^), (**d**) NPP (g C m^−2^ yr^−1^), (**e**) LAI (m^2^ m^−2^), (**f**) WUE (g C kg^−1^ H_2_O), and (**g**) uWUE (g C Pa^0.5^ kg^−1^ H_2_O) over China during 2003–2018

Figure 1 shows the annual mean meteorological and vegetation conditions over China based on 2003–2018 observations. The annual mean precipitation over China is 604 mm yr^−1^ with significant spatial heterogeneity (Fig. 1a), with a wide range of values from < 100 to 2290 mm yr^−1^. The highest precipitation is mainly distributed over southeastern China where abundant water vapor is transported from the south by the summer monsoon. The mean soil moisture derived from ERA5 over China is 0.27 m^3^ m^−3^, with very dry soil conditions over northwestern China (Fig. 1b). Soil moisture is not only related to accumulated precipitation but also to changes in evapotranspiration, which indicates the dynamics of the water budget.

The mean GPP over China is 511 g C m^−2^ yr^−1^ based on MODIS (Fig. 1c). The maximum GPP is concentrated over southeastern China and the minimum GPP is mainly distributed over northwestern China. The mean NPP is 345 g C m^−2^ yr^−1^ over China (Fig. 1d). Forests are the dominant vegetation type for southeastern China, whereas northwestern China consists of barren land and grasslands with relatively low vegetation productivity. The spatial pattern of LAI (Fig. 1e) is similar to GPP and NPP, with a mean LAI of 0.93 m^2^ m^−2^. The mean annual WUE and uWUE of China’s terrestrial ecosystems during 2003–2018 are 0.8 g C kg^−1^ H_2_O and 20 g C Pa^0.5^ kg^−1^ H_2_O, respectively. WUE is higher for southern and northeastern China mainly covered of forests, and lower over western China and Tibetan Plateau due to the low productivity and high demand of ET (Fig. 1f) [28]. The mean annual uWUE is different from WUE over northeastern and northern China (Fig. 1g). Southern China has higher uWUE, suggesting higher ratio of vegetation photosynthesis to stomatal conductance regardless of the influence of VPD (Fig. 1g).

### Characteristics of flash drought events and the related meteorological conditions over China

**Fig. 2:**
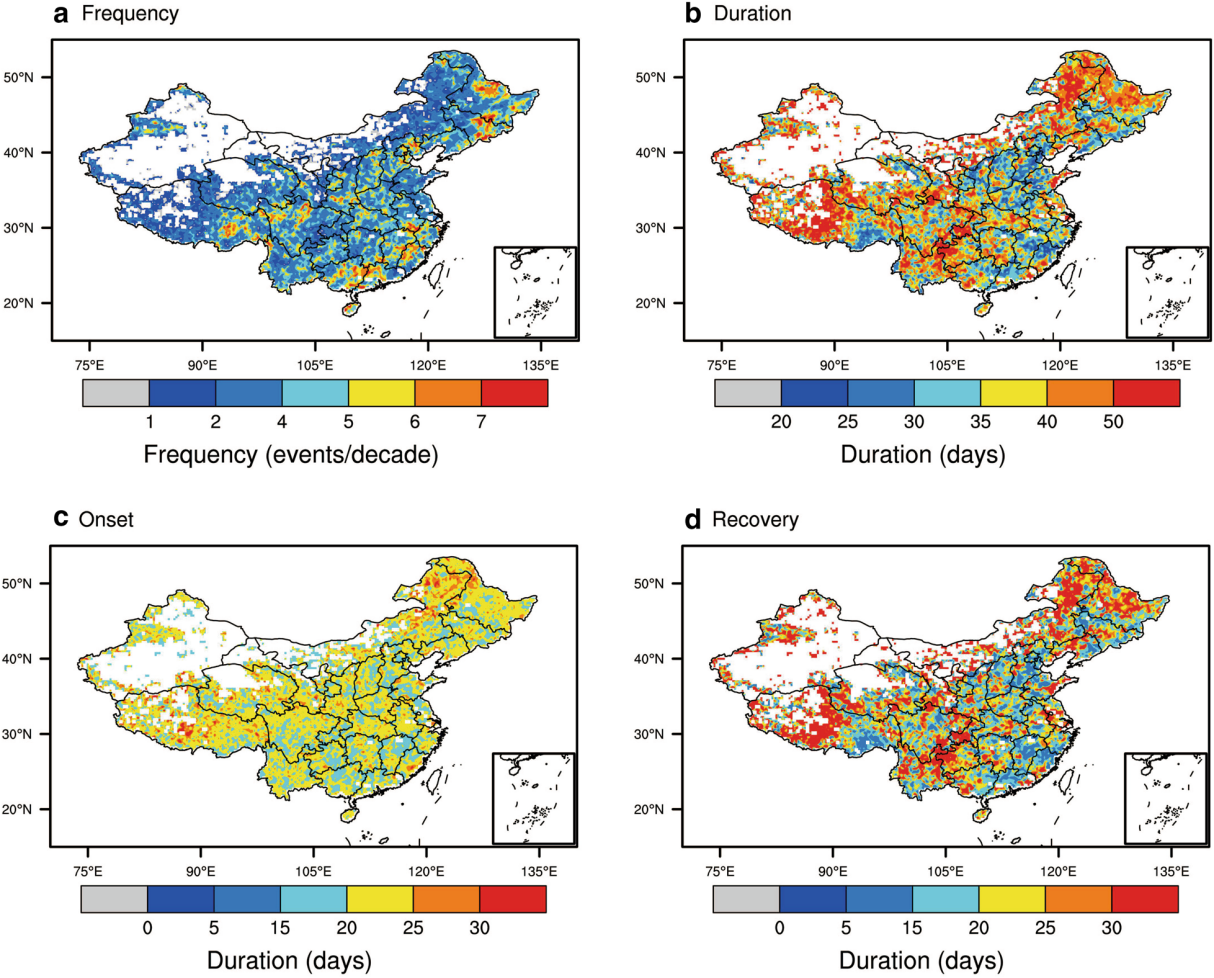
Frequency and mean duration of flash droughts (**a**–**b**), and the duration of onset and recovery stages (**c**–**d**) over China during 2003–2018

Figure 2 shows the frequency and mean duration of flash droughts over China during 2003-2018, as well as the mean duration of their onset and recovery stages. The mean frequency of flash droughts is 2.3 events per decade across all of China, with some hot spots over southeastern China and northeastern China. The mean duration averaged over China is 43 days, with the longest average durations located over northeastern and western China. The mean annual precipitation over the areas with relatively long flash droughts is usually lower than 500 mm yr^−1^ for Tibetan Plateau and northeastern China (Fig. 1a). The mean durations of the onset and recovery stages are 21 days and 23 days, respectively. For the duration of flash drought onset stages, there is no significant difference over different climate regions (Fig. 2c). The duration of the recovery stage is longer in parts of the Tibetan Plateau, southwestern and northeastern China (Fig. 2d). The mean duration of recovery is 23.7 days for northeastern China and the recovery stage of flash droughts could last for even several months.

**Fig. 3:**
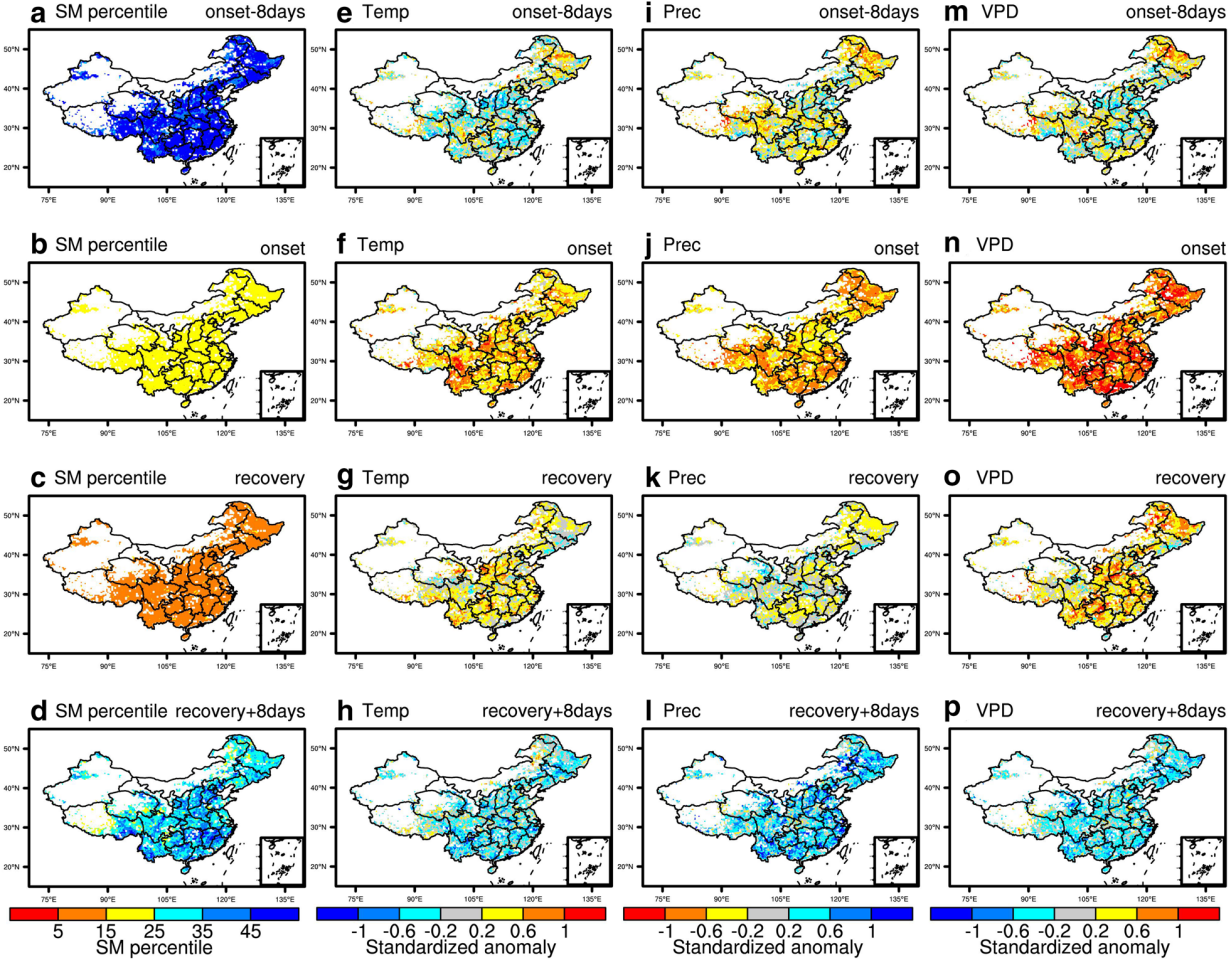
Hydrometeorological conditions during 8 days prior to flash drought, onset and recovery stages of flash drought, and 8 days after flash drought. (**a**–**d**) soil moisture percentiles, standardized anomalies of temperature (**e**–**h**), precipitation (**i**–**l**), and VPD (**m**–**p**)

Figure 3 shows the meteorological conditions and soil moisture percentiles during 8 days prior to flash droughts, the onset stage, the recovery stage, and 8 days after the recovery stage. Only the grid points with at least 3 flash drought events identified from 2003 to 2018 are shown. The soil moisture percentile during 8 days prior to the onset of flash droughts is close to the 49% percentile (Fig. 3a). There is then a quick transition to much drier conditions, where the mean soil moisture percentile is 19% during the onset stage (Fig. 3b). After the rapid onset of drought, the decreasing rate of soil moisture slows down and the mean soil moisture percentile is 11% during the recovery stage (Fig. 3c). The soil moisture recovers quickly to the 32nd percentile after the end of flash drought. During the onset stages of flash droughts, the anomalies are 0.49, − 0.58, and 0.81 for standardized temperature, precipitation, and VPD respectively, indicating that they are characterized by well below normal precipitation and elevated evaporative demand. The increase in precipitation and decreased VPD relieve the soil moisture condition after the end of flash drought.

### Responses of GPP, NPP, and LAI to flash drought over China

**Fig. 4:**
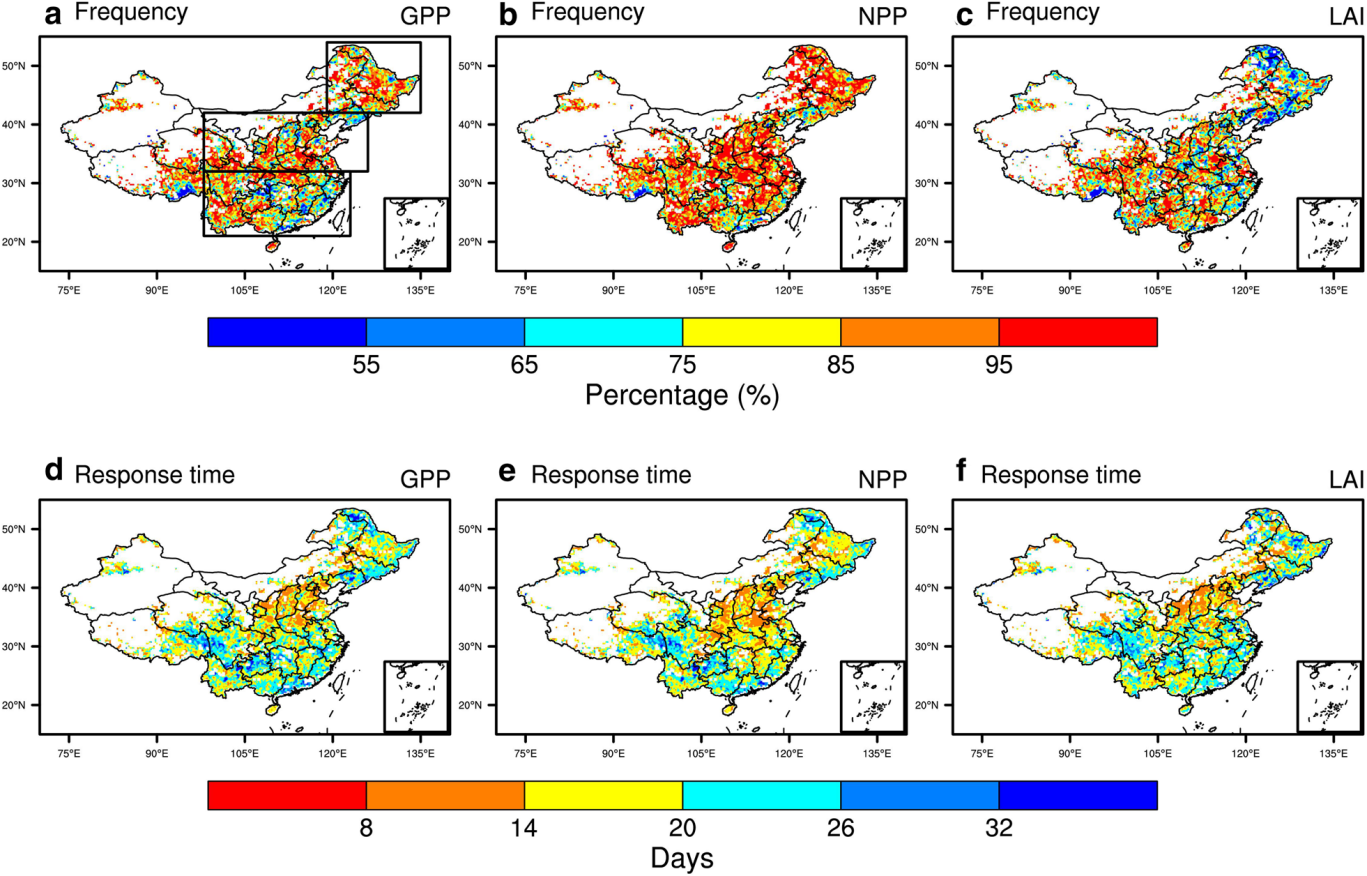
The response frequency (**a**–**c**) and response time (**d**–**f**) of GPP, NPP, and LAI to flash droughts over China. The response frequency is the ratio of flash droughts with negative GPP (NPP/LAI) anomalies to the total flash drought events. The response time is the first occurrences of negative GPP anomalies during flash drought. The boxes are the locations of southern China (21–32 °N, 98–123 °E), northern China (32–42 °N, 98–126 °E), and northeastern China (42–54 °N, 119–135 °E)

**Fig. 5:**
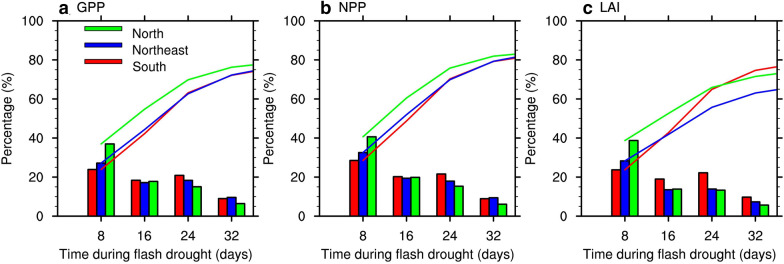
Percentage of response time of (**a**) GPP, (**b**) NPP, and (**c**) LAI to flash drought for the northern (green), northeastern (blue), and southern China (red). Bars represent the percentages at different response time and lines are accumulated response percentages at certain response time

Figures 4a–c show the frequencies of flash drought with the occurrences of negative anomalies of GPP, NPP, and LAI during flash drought. There are substantial differences in the response frequencies for GPP, NPP and LAI. The response frequencies are 81%, 86%, and 79% for GPP, NPP, and LAI, respectively. For northeastern China, negative LAI anomalies only occur during 74% of the identified flash droughts, which is much lower than 84% for GPP and 90% for NPP. Photosynthesis is not only related to the change of LAI but also physiological processes (e.g., enzyme activity), therefore the response of GPP and NPP to flash drought is more significant than LAI. In terms of the response time to flash droughts, the distributions of response time based on GPP, NPP, and LAI over China are quite similar. And the mean response time over China is around 19 days. However, the response time of GPP and LAI for northern China is 16 days with negative anomalies occurring during more than 50% of the flash drought events, which is much quicker than 24 days of southern and northeastern China (Fig. 5). The frequencies of response time are similar for GPP and NPP (Fig. 5a, b), and the lag time between the onset of flash drought and the subsequent ecological response is shorter over northern China than that over southern and northeastern regions. For northern China in semiarid region, water availability dominates the carbon cycle and the ecosystem is more sensitive to drought [38]. The response of LAI increases rapidly after 16 days of flash drought over southern China (Fig. 5c).

### Response of WUE to flash drought over China

**Fig. 6:**
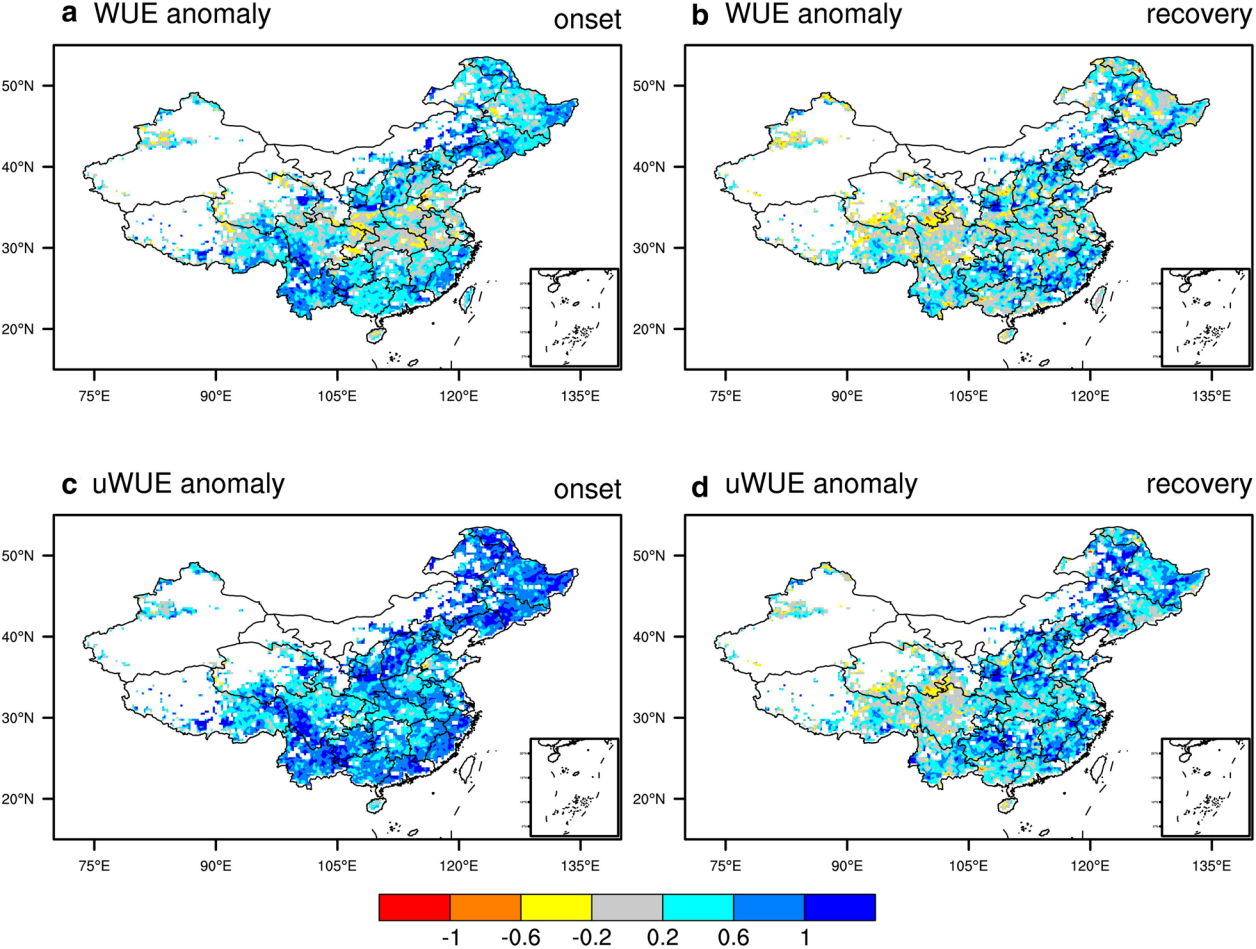
Standardized anomalies of water use efficiency (WUE) and underlying WUE (uWUE) during onset and recovery stages of flash drought

Figure 6 shows the standardized anomalies of WUE and uWUE over the onset and recovery stages of flash drought. During the onset stages, the increase of uWUE is 0.67, which is more obvious than 0.36 of WUE and the difference is mainly attributed to the impact of higher VPD. Besides the effects of drought on WUE, higher VPD could increase potential ET and enhance water loss. The increase in WUE and uWUE shows the vegetation resilience to drought, whereas the magnitudes of their increase are reduced during recovery stages. The anomalies of WUE and uWUE during the recovery stages are 0.24 and 0.42, respectively. The adaptation of vegetation to drought decreases with increasing duration of drought. However, there is no significant increase in WUE and uWUE in western China during the recovery stages of flash droughts.

## Discussion

The rapid change of soil moisture conditions is triggered by high VPD and large precipitation deficits. The rapid intensification of soil moisture droughts leaves limited time for taking measurements [39]. The deficits in soil moisture could decrease stomatal conductance to avoid additional water loss. In the meantime, the diffusion of CO_2_ from the atmosphere into the leaf is also reduced. Besides, the decrease in atmospheric humidity further causes plants to close their stomata. The combined atmospheric and soil conditions during flash droughts have synergistic effects on vegetation photosynthesis and transpiration processes thus altering the coupling between carbon and water fluxes. The response of ecosystem respiration to flash droughts is not only sensitive to soil moisture droughts, but also to higher temperatures.

The ecological response of NPP occurs during 86% of the flash drought events, whereas GPP and LAI are less sensitive to flash droughts. The discrepancy between the response frequencies of GPP and NPP is due to the role of vegetation respiration in the terrestrial carbon sink. Vegetation respiration is less affected by drought conditions than photosynthesis [40], and the greater reduction in NPP may be correlated with the higher ratio of respiration to GPP during drought [11]. Besides, the positive anomalies of temperature during flash droughts might also increase ecosystem respiration and further intensify the carbon loss of terrestrial ecosystem [29]. He et al. [20] found that the enhanced respiration caused more carbon loss for grasslands and croplands during the 2012 flash drought over North America. The responses of LAI occur during 79% of the flash droughts, indicating the structural changes of the ecosystem during flash droughts. Besides the decrease in LAI, vegetation photosynthesis is also suppressed by the reduction of canopy conductance induced by drought [18], which is consistent with higher sensitivity of GPP and NPP to drought than LAI [41]. The discrepancies for the response frequencies of different ecological metrics are largest for northeastern China.

The mean response time averaged over China is 19 days for GPP, NPP, and LAI, with quicker response over northern China that has a semiarid climate. Soil moisture plays a dominant role in ecosystem productivity for northern China, and the responses of GPP and LAI in more than 50% of flash drought events occur within 16 days, prior to those for southern China and northeastern China. The response of LAI increases rapidly during 17–24 days of flash drought for southern China, which is comparable to northern China. In addition, the accumulated impact of flash droughts on the ecosystems may also be related to the characteristics of flash droughts including severity and duration [42, 43], and the longer flash droughts would cause more carbon loss of terrestrial ecosystems. Although the soil moisture drought recovers, it needs time for vegetation to recover to its normal condition.

Higher WUE and uWUE during flash droughts show the resistance of ecosystems to flash drought. However, the response of WUE and uWUE differ with the increase in drought duration. The standardized anomalies of uWUE are 0.67 and 0.42, respectively, for the onset and recovery stages. However, the increase in WUE is less compared with the positive anomalies of uWUE under the influence of VPD. Higher VPD during flash droughts would decrease WUE due to water loss driven by high atmospheric demand. During the recovery stage, there are no significant changes in WUE and uWUE over western China, indicating a more vulnerable ecosystem condition of grasslands in western China than forests in southern and northeastern China.

In this study, we used top 1-m soil moisture from ERA5 as root-zone soil moisture for all of China. Considering that some plants have roots deeper than 1-m, we also analyzed the response of the ecosystem to flash droughts identified by anomalies in 2-m soil moisture. The mean frequency of flash droughts identified by 2-m soil moisture is 1.7 events per decade, which is less than the 2.3 events per decade identified using 1-m soil moisture. The mean duration of flash droughts is longer for 2-m soil moisture. However, the frequencies of flash droughts causing decline in GPP, NPP, and LAI range from 80% to 87%, and the response time of GPP, NPP, and LAI is around 19 days, which are similar to the results based on 1-m soil moisture (not shown). Therefore, it is reasonable to use 1-m soil moisture to analyze the impacts of flash droughts. On the other hand, although remote sensing provides regional observations of vegetation conditions, the direct impacts of soil moisture on GPP is underestimated [44] and it still needs more effort using multiple observations to analyze the drought impacts on terrestrial ecosystems.

## Conclusion

This study shows the rapid response of ecosystems to flash droughts using multiple ecological metrics derived from MODIS. The response of GPP, NPP, and LAI show the reactions of ecosystems to flash droughts from vegetation physiological and structural perspectives. The results show that NPP is more sensitive than GPP and LAI to the occurrence of flash drought, with differences attributed to the vegetation respiratory process and physiological process of photosynthesis. The ecological response to flash drought is quicker for northern China containing a semiarid climate than it was for more humid portions of the country. However, it is still necessary to improve the satellite observations of ecosystem productivity [44], and modeling method would benefit from the study of the mechanisms of drought impact on ecosystems [45]. This study suggests the possibility of using satellite observations to detect the ecological impacts of flash droughts.

## Data Availability

The soil moisture and ET dataset is derived from ERA5 through https://cds.climate.copernicus.eu/#!/search?text=ERA5&type=dataset. MODIS GPP, NPP, and LAI are available for public use (https://modis.gsfc.nasa.gov/). The daily precipitation, temperature, and relative humidity are from China Meteorological Data Service Center (http://data.cma.cn).

## Abbreviations

MODIS: Moderate Resolution Imaging Spectroradiometer
ERA5: European Centre for Medium-Range Weather Forecasts Reanalysis 5
USDM: United States Drought Monitor
NASA: The U.S. National Aeronautics and Space Administration
SM: Soil moisture
ET: Evapotranspiration
GPP: Gross primary productivity
NPP: Net primary productivity
LAI: Leaf area index
WUE: Water use efficiency
uWUE: Underlying water use efficiency
VPD: Vapor pressure deficit
PAR: Photosynthetically active radiation
fPAR: Fraction of photosynthetically active radiation
